# Personal Exposure to Fine Particles (PM_2.5_) in Northwest Africa: Case of the Urban City of Bamako in Mali

**DOI:** 10.3390/ijerph19010611

**Published:** 2022-01-05

**Authors:** Alimata Sidibe, Yosuke Sakamoto, Kentaro Murano, Ousmane A. Koita, Ibrahim Traore, Yacouba Dansoko, Yoshizumi Kajii

**Affiliations:** 1 Graduate School of Global Environmental Studies, Kyoto University, Yoshida-Honmachi, Sakyo-ku, Kyoto 606-8501, Japan; sakamoto.yosuke.7a@kyoto-u.ac.jp (Y.S.); murano@hosei.ac.jp (K.M.); kajii.yoshizumi.7e@kyoto-u.ac.jp (Y.K.); 2 Graduate School of Human and Environmental Studies, Yoshida-Nihonmatsu-Cho, Sakyo-ku, Kyoto 606-8501, Japan; 3 Regional Environment Conservation Division, National Institute for Environmental Studies, Tsukuba 305-8506, Japan; 4 Laboratoire de Biologie Moléculaire Appliquée, Faculté des Sciences et Techniques (FAST), University of Sciences, Techniques and Technologies of Bamako (USTTB), Bamako E 3206, Mali; okoita@icermali.org (O.A.K.); ibrahim.traore@lbma.edu.ml (I.T.); yacoubadansoko@yahoo.fr (Y.D.)

**Keywords:** Bamako, air pollution, PM_2.5_, daily activities, health, mitigation strategies

## Abstract

Personal exposure to particulate matter (PM) from anthropogenic activities is a major concern in African countries, including Mali. However, knowledge of particulates is scant. This study was undertaken to characterize personal exposure to PM_2.5_ microns or less in diameter (PM_2.5_) in the city of Bamako in Mali. The exposure to PM_2.5_, through daily activities was observed from September 2020 to February 2021. Participants wore palm-sized optical PM_2.5_ sensors on their chest during their daily activities. The exposure levels in four different groups of residents were investigated in relation to their daily activities. The variation in PM_2.5_ concentration was measured during different activities in different microenvironments, and the main sources of exposure were identified. The highest average 10 min concentrations were observed at home and in bedrooms, while the participants were using specific products typically used in Africa, Asia, and South America that included insecticides (IST; 999 µg/m^3^) and incense (ICS; 145 µg/m^3^), followed by traffic (216 µg/m^3^) and cooking (150 µg/m^3^). The lowest average 10 min concentrations were also observed in the same microenvironment lacking IST or ICS (≤14 µg/m^3^). With no use of specific products, office workers and students were the least exposed, and drivers and cooks were the most exposed. The concentrations are up to 7.5 and 3 times higher than the World Health Organization’s yearly and daily recommended exposure levels, respectively, indicating the need to promptly elaborate and apply effective mitigation strategies to improve air quality and protect public health. This study highlights the importance of indoor air pollution sources related to culture and confirms previous studies on urban outdoor air pollution sources, especially in developing countries. The findings could be applied to cities other than Bamako, as similar practices and lifestyles are common in different cultures.

## 1. Introduction

Particulate matter (PM) is one of the most widespread pollutants in the atmosphere and has attracted the interest of numerous air-quality researchers. PM exists in the atmosphere as solid or liquid suspensions. They originate from natural sources, volcanic eruptions, sea salt, wildfires, and anthropogenic sources, like road traffic, biomass combustion, and waste incineration. They can be dispersed by wind. When directly emitted from pollution sources, particles are referred to as primary PM. Secondary PM is produced by the reaction of primary precursor gases entering the atmosphere. Particle sizes differ from a few nanometers to hundreds of micrometers. They are classified according to size from smallest to largest as the Nuclei mode (ultrafine fraction), Aitken mode (fine fraction), Accumulation mode, and Coarse mode. The mass distribution is dominated by the Accumulation and Coarse modes [1].

Depending on their size, the various PM types have multiple harmful effects on the environment and human health. Smaller particulate matter can undergo long-range transport and, in addition to local impacts, generate regional and global impacts. They are most involved in atmospheric reactions by natural processes. They directly affect climate change through their role as cloud condensation nuclei [1] and reduce visibility in the troposphere by scattering solar radiation [2]. Worldwide, many studies have provided evidence of the effects of PM on human health, especially the fine and ultrafine fractions, as they can reach the deepest regions of the respiratory system. Short-term variation and levels of urban particulate air pollution are associated with increases in lung function deterioration, respiratory diseases, number of hospital admissions, and mortality from cardiorespiratory problems and cancers [3]. Countries including China, India, France, Italy, and the United States have reported health issues related to exposure of PM_2.5_ microns or less in diameter [4,5,6,7]. However, information is scant in developing countries.

In Africa, air pollution has reached a significant scale. The average concentration of PM_2.5_, ranging up to 507 µg/m^3^, has been recorded in multiple African cities. The concentrations exceed the World Health Organization Air Quality Guideline (WHO AQG) in nearly all African cities where PM_2.5_ data are available [8]. Rapid population growth has led to the increased use of natural resources and the emission of chemical molecules that affect ecosystems. Air pollution in Africa and the resulting health effects are strongly related to socioeconomic status. The majority of risk factors, such as biomass combustion, transportation (vehicle age, motorcycle, taxis, and buses), unpaved roads, and street food preparation are more pronounced [9,10]. For instance, the majority of households in developing countries burn biomass fuels in open fireplaces, and the car fleets are older, poorly maintained, and use low-quality fuels with high lead concentrations, which generate high levels of pollutants [11]. Household air pollution from solid fuels contributes to ambient particulate matter pollution and has been identified as the second disease burden in most of sub-Saharan Africa, and the fourth globally [12,13]. To overcome these issues, international institutions have established international development goals in sustainable development (SDGs).

The vehicle fleet is predominately old in the city of Bamako, located in Mali in West Africa, as well as in many other developing countries that include Lagos in Nigeria [8], and Addis Ababa in Ethiopia [14]. Legislation on the importation of low-quality fuels that are potential sources of major pollutants including nitrogen dioxide (NO_2_) and sulfur dioxide (SO_2_) is extremely lax. For instance, the importation of diesel fuel with a sulfur content of 10,000 ppm is legal in Mali, while the threshold allowed in Europe is only 10 ppm [15]. Recorded NO_2_ and SO_2_ values of 60 µg/m^3^ and 29 µg/m^3^, respectively, in traffic sites in Bamako exceed World Health Organization (WHO) guidelines [8]. In addition, waste incineration is the most widely used waste management technique. This, releases considerable quantities of fine particles and other pollutants into the atmosphere. Wood and charcoal are resources that account for 78% of the national energy balance [16]. They are extensively used in households and are a major source of PM emissions. In addition, the combustion of insecticides and incense, which produce a considerable amount of PM, is widespread. The city of Bamako is surrounded by hills [17], and so is poorly ventilated. Consequently, the city is subject to stagnant pollutants from the diverse aforementioned anthropogenic sources. The number of patients with respiratory diseases has increased in Bamako during the last few years [18].

There are no published data concerning population exposure to PM_2.5_ particulate matter in Bamako. The present study addressed this. Personal exposure to PM_2.5_ has been investigated in four different groups of local inhabitants. Participants wore a newly developed palm-sized particle sensor (PM_2.5_ sensor) that was positioned in a lanyard on the chest. The PM_2.5_ sensor is portable, light, inexpensive, has low energy consumption and good data storage capacity, and is reliable. Nakayama et al. (2018) described the performance and characteristics of sensors [19].

The data obtained from the present study are essential to inform the local population about their exposure to PM_2.5_ through daily activities and help increase awareness regarding adverse health effects. Additionally, the data could help to better understand the relationship between the control of environmental problems and promoting sustainable development. This study could provide the local government with reasonable strategies to reduce air pollutant emissions. Finally, this study provides background information on PM_2.5_ exposure concentration in this region and can guide further studies on air pollution in Mali.

## 2. Materials and Methods

### 2.1. Sampling Location

**Figure 1:**
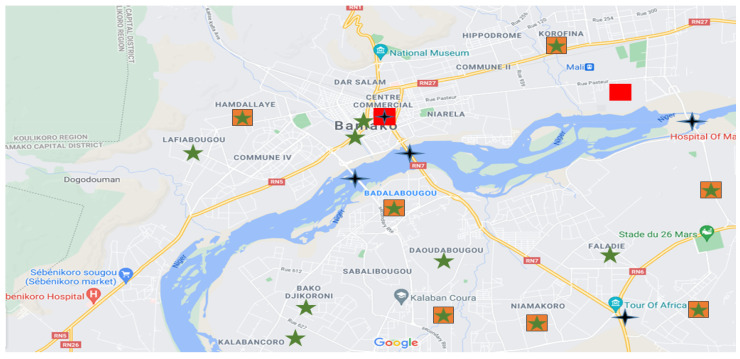
Different microenvironments’ locations of the participants on Google Maps; green star (houses), orange square (work places), black star (high traffic areas), red square (industrial zones).

PM sampling was performed within five months from September 2020 to February 2021 in different microenvironments in Bamako city (Figure 1). This urban city is the capital of the West African country of Mali. It is located in southwest Mali (12°39′0.00″ N–8°00′0.00″ W). The population is approximately three million [20]. The major possible sources of particles in this region are combustion from biomass and biofuel usage, soil dust resuspension, road vehicle tires, traffic, waste incineration, and natural processes, such as harmattan occurring during the dry season [21].

### 2.2. Materials

**Figure 2:**
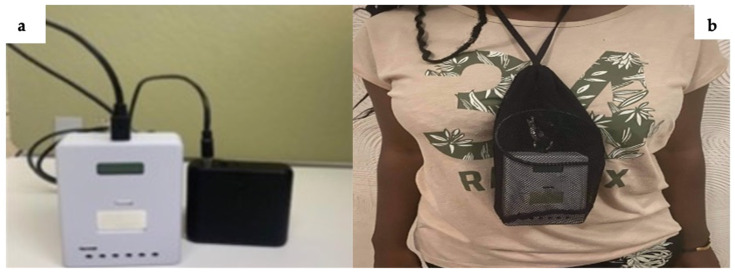
The P-sensor (**a**) and its typical orientation (**b**).

To obtain information about personal exposure, each participant wore a palm-sized optical PM_2.5_ sensor (P-sensor; Figure 2a). The palm-sized sensor was specifically designed to give the mass concentration (in μg/m^3^) of particulate matters of 2.5 microns or less in diameter. A P-sensor works based on the principle of heat convection transfer and light-scattering of the particles. A heater inside the instrument directs the particles toward a light-emitting diode, where they will be irradiated and will scatter lights with intensities proportional to their sizes [19]. According to the specifications, the P-sensor is optimized to measure particles >10 µg/m^3^. Therefore, lower values would be less reliable [22]. In addition to real-time personal exposure sampling, each participant used a time diary to indicate their locations and activities during the sampling period.

### 2.3. Sampling Procedure

Personal exposure to PM_2.5_ was measured in participants who lived in Bamako using palm-sized portable PM_2.5_ sensors. The participants were selected based on major occupations in the city: office workers (OW), drivers (DRI), cooks (COOK), and students (ST). Participants in these occupations were selected with no restrictions on gender, age, area of residence, house conditions, and personal habits (smoking, exercising, etc.). Each participant provided personal information after agreeing to take part in the research. For each occupational group, at least three participants were sampled for 3 days. The sensor was worn around the neck on the chest, close to the breathing zone (Figure 2b). The real-time mass concentrations of PM_2.5_ were recorded at 5 s intervals. The sensor was removed during bathing, sleeping, and battery charging. At those times, it was placed on a support located in the same microenvironment. Each participant recorded their daily activities in a diary, describing the specific activities they performed and the respective microenvironments. They gave notice whenever they switched their microenvironment or activities. The diaries provided information on the time, location, and activities of the participants for 72 h. The time and location were characterized by different activities and microenvironments indoors (such as home, home with incense and insecticides, workplace, classroom, other activities) and outdoors (such as market, cooking, driving, other activities). PM_2.5_ exposure data for a participant’s total exposure were then obtained from measurements in various locations during the sampling period. The participants’ information (occupation, gender, age, mean of transportation, and personal habits) was collected using Table S2.

### 2.4. Quality Assurance and Control

The palm-sized PM_2.5_ was evaluated by its developers using two types of standard beta attenuation monitors (DKK-TOA, model FPM-377 and Kimoto, model PM-712) at four different locations in Japan (Fukuoka, Kadoma, Kasugai, and Tokyo). The daily averaged mass concentration obtained from the PM_2.5_ sensors were in good agreement with the standard instruments (with R between 0.89 to 0.95) [19].

**Figure 3:**
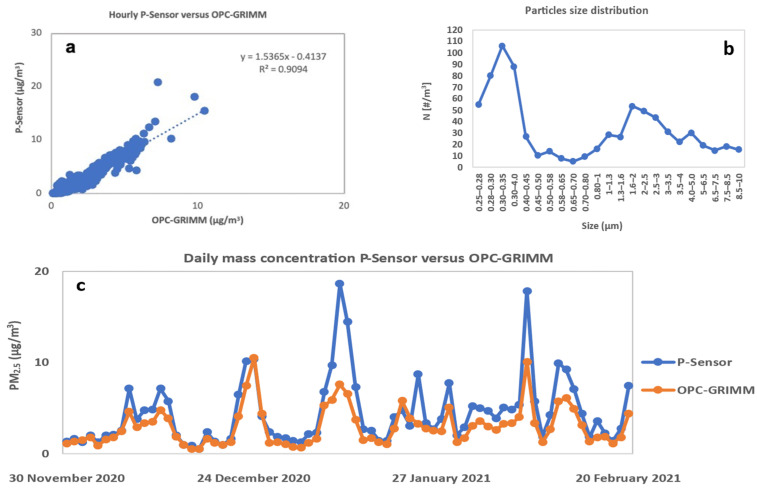
(**a**) Correlation plots of the P-sensor versus the GRIMM OPC from 30 November to 22 December 2020, and from 7 January to 20 February2021; hourly average concentration. (**b**) Typical size distribution of PM from the GRIMM OPC. (**c**) Daily variation of PM_2.5_ concentration for the GRIMM OPC versus the P-sensor.

Furthermore, we tested the performance of the palm-sized PM_2.5_ sensor by comparing it to a Grimm model 1.109 optical particle counter (OPC). Sampling was performed at Kyoto University, Yoshida South Campus (Figure S2) from 1–22 December and from 7 January to 20 February. The particle number distribution over 250 nm in diameter by the Grimm OPC covered >80% of the volume fraction and was converted into mass concentration using a typical mass density of ambient particles of 1.2 g cm^−3^ [23,24,25]. The typical size distribution of PM provided by OPC Grimm showed that the mass concentration of PM was dominated by PM_2.5_ (Figure 3b). The results showed an agreement in the time variation (Figure 3c) and a good correlation between the two instruments (R = 0.90 and 0.82 for the 1 h and 24 h average, respectively; Figure 3a and Figure S1b). This correlation coefficient was similar to the one obtained by Nakayama et al. in 2018 [19]. Additionally, data obtained from the Japanese Ministry of Environment’s website (AEROS) available at (http://soramame.taiki.go.jp/, accessed on the 26 June 2021) were used for comparison [26]. The results showed good agreement in time variation between Yamashina station (5.8 km from Kyoto University, Yoshida South Campus) and the P-sensor from 16 November to 20 February (Figure S1a).

To ensure the quality of the data collected on personal exposure to PM_2.5_, the participants were given lectures to explain to them the content of the study, as well as its objectives and methods prior to data collection. They were instructed on how to record detailed information during the sampling period. The lecture also included demonstrations on how to operate the sensors. Each participant was given a chance to operate a sensor before the official start of the samplings. The communication between the participants and the research team was constant during the sampling period so that any problem or question could immediately be addressed. Batteries were regularly charged during sleep time without interrupting the samplings, to prepare for the next day. This was possible, as the batteries have multiple connection ports. Data were downloaded and saved from the sensors after each participant completed the required sampling period. Sensors and batteries were checked for any malfunction before assigning them to the next participants. Only data obtained from participants providing clear information on time, location, and activity were used for the data analysis.

### 2.5. Data Analysis

The diaries and the recorded concentrations from the devices were used to calculate personal exposure to PM_2.5_. Background concentrations were considered as concentrations recorded in absence of local emission sources. These concentrations were very low compared to those recorded during the participants’ daily activities, and were included in our calculations.

#### Personal Exposure Calculation Method: Case of the Office Workers

The personal exposure to PM_2.5_ was calculated using the recorded concentrations for different activities and microenvironments along with the time spent for these activities and microenvironments.

(1) $$OW1d_{1}=\frac{\sum_{i=1}^{n}OW1C_{1i}t_{1i}}{24}$$

where *OW*1*d*_1_ is the average exposure of office worker 1 for day 1(*d*_1_). $\sum_{i=1}^{n}OW1C_{1i}t_{1i}$ represents the integrated exposure concentration of the office worker on *d*_1_. It was obtained from the product of the total average concentration (μg/m^3^) recorded in all the microenvironments (*C*_1*i*_) on *d*_1_, such as home, office, transportation, and stores, and the time (hours) spent in the microenvironments (*t*_1*i*_). Note that 24 is one day in hours.

The average exposure of all office workers was calculated as follows:

(2) $$AvgOW=\frac{\left(OW1d_{1}+OW1d_{2}+OW1d_{3}+\ldots +OW2d_{1}+OW2d_{2}+\ldots +OWnd_{3}\right)}{N}$$

where *OW* is the group of office workers, and *OWn* represents the total number of office workers. *d*_1_, *d*_2_, *d*_3_ are sampling days 1, 2, and 3. *N* is the number of office workers multiplied by the number of sampling days.

The same method was applied to obtain the exposure concentrations for the group of students, cooks, and drivers.

## 3. Results

### 3.1. Daily Time-Series of PM_2.5_ Concentration

Although the data presented in this study were collected during the COVID-19 pandemic, we presumed that the pandemic did not influence the results. The restrictions for coronavirus case reduction strategies were no longer applicable in Bamako. Citizens had already resumed their daily activities. Consequently, outdoor emission sources remained unchanged. Furthermore, household activities (house chore, cooking and other combustion sources) were sustained as they were not related to the COVID-19 restrictions in Bamako.

#### 3.1.1. OW

**Figure 4:**
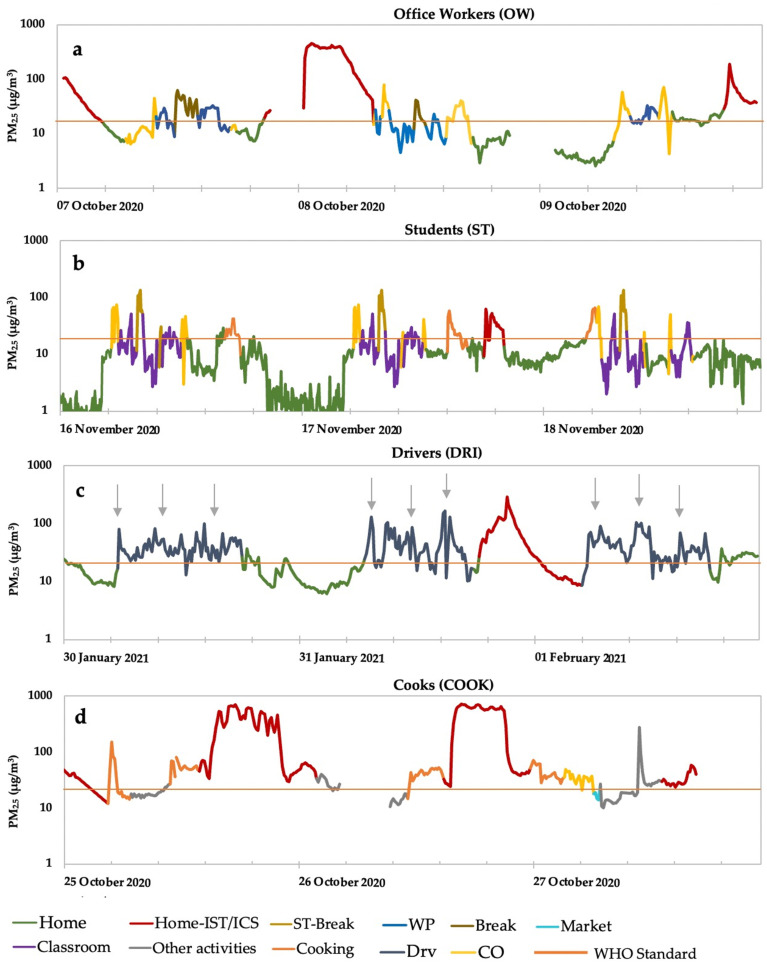
Typical daily personal profile adopted from OW2 (**a**), ST1 (**b**), DRI3 (**c**), and COOK1 (**d**) between September 2020 and February 2021. WHO: World Health Organization, IST/ICS: insecticide/incense, OW: Office workers, ST: Students, DRI: Drivers, WP: Workplace, Drv: Driving, CO: Commute. The grey arrows indicate the rush hours.

Five participants were OW. Figure 4a shows a typical PM_2.5_ concentration variation by OW2 between 7–9 October 2020. The air conditions of OW were mainly categorized as home, commuting, office, and home (IST/ICS). OW2 was exposed to a 3-day average of 50 µg/m^3^ with daily concentrations of up to 102 µg/m^3^. The green color in the figure represents the PM_2.5_ concentration at home with an average value of 10 µg/m^3^ and a maximum value of 29 µg/m^3^. The recorded concentrations during the commute to work of OW2 averaged 21 µg/m^3^ with a maximum of 79 µg/m^3^. The concentration in the office, represented by the blue color, averaged 16 µg/m^3^ with a maximum of 33 µg/m^3^. A higher average concentration of 34 µg/m^3^ was recorded during the participant’s break time, represented in brown in the figure.

The red color in the figure shows concentrations at home when the participant was using insecticides (IST) and incense (ICS), which are popular in the region. The average and maximum values were 163 µg/m^3^ and 460 µg/m^3^, respectively. The home air with the use of insecticides and incense was separately categorized from the period when these products were not used, due to large differences in concentration. ICS is widely used in Bamako to provide a pleasant scent in the home and is part of the Malian culture. IST are used to prevent mosquito bites and reduce the risk of malaria. These products were typically used every day by the participants. As shown in Figure 4a, the highest exposures to OW2 were observed during the use of IST/ICS and the commute.

#### 3.1.2. ST

Figure 4b shows the daily representative PM_2.5_ concentration variations for one individual (ST1) from 16–18 November 2020. The microenvironments and activities describing air conditions for ST1 were mainly home, commuting, school, break time at school (outside), and home (ISC/IST). The green color represents the concentration of PM_2.5_ at home (average concentration 8 µg/m^3^). The participant’s room was located near the kitchen, which increased the average concentration from 8 to 29 µg/m^3^ during cooking, represented in orange. Yellow indicates the concentration during the commute (walking or driving) between home and school, which averaged 37 µg/m^3^. Heavy-traffic areas emitting high levels of PM_2.5_ have a higher health impact than other pollutants on pedestrians [27]. Purple indicates the concentration at school during classes, which averaged 14 µg/m^3^. The highest concentration for ST1 was observed during break time, with an average and maximum value of 80 and 133 µg/m^3^, respectively. The high concentration during break time can be explained by soil dust resuspension, as the schoolyard was dry (Figure S6). The use of ICS is represented by a red color. The average and maximum concentrations were 34 and 62 µg/m^3^, respectively. The daily PM_2.5_ concentrations in this group were affected by the commute and break time. The findings indicate high exposure to PM_2.5_ both indoors and outdoors for the ST group.

#### 3.1.3. DRI

Two microenvironments and one activity were categorizing air conditions for public transportation drivers: home, driving, and home (IST/ICS). These are represented in green, gray, and red (Figure 4c). The daily representative PM_2.5_ concentration variations for a public transportation driver (DRI3) from 30 January 2020 to 1 February 2021, are presented. DRI3 was exposed to 14 µg/m^3^ on average at home, increasing to 43 µg/m^3^ on average during working hours corresponding to driving. The concentration reached 167 µg/m^3^ during rush hours, generally between 7:00 to 9:00 a.m., 11:30 a.m. to 1:00 p.m., and 3:30 to 7:00 p.m. (gray arrows). The highest concentration was 81 µg/m^3^ on average with a maximum of 290 µg/m^3^ recorded at bedtime after the use of insecticides. DRI3 was highly exposed to PM_2.5_, for a considerable amount of time.

#### 3.1.4. COOK

Activities of COOK were limited to food preparation and other household activities, such as cleaning the house, doing dishes, and groceries, indicating the exposure level in this group. Regular daily PM_2.5_ concentration variations are shown in Figure 4d, adopted from COOK1 from 25–27 October 2020. COOK1 was exposed to a 3-day average concentration of 128 µg/m^3^. The highest average concentration was observed at home during sleep (228 µg/m^3^). The concentrations during such events reached very high peaks of up to 717 µg/m^3^. Cooking time, shown in orange, represented the second-highest average concentration of 42 µg/m^3^ with a maximum of 150 µg/m^3^. A high concentration of 27 µg/m^3^ on average was also observed when COOK1 performed other household activities (gray color). The use of specific products, charcoal, and wood as cooking fuel and a cooking stove not adapted for efficient combustion were sources of exposure to a high concentration of particles during the entire sampling period.

### 3.2. Comparison of Different Groups

Table 1 summarizes the PM_2.5_ concentrations of the participants’ daily activities averaged for each group. Information about the concentration for each group is provided in separate tables (Tables S3–S6) in the Supplementary Materials.

The highest concentration of 207 µg/m^3^ on average was observed at home with IST/ICS usage, followed by cooking (42 µg/m^3^), driving and commuting (35 µg/m^3^), school (Break; 31 µg/m^3^, beauty salon (19 µg/m^3^), school 16 µg/m^3^), and home without IST/ICS usage (13 µg/m^3^). In this study, concentrations in the same microenvironments and activities were similar, even in different groups (Table 1).

IST/ICS average values from this study (207 ± 115 µg/m^3^) are comparable to the average value reported by Kumar (2014) in India (256.8 µg/m^3^) [28]. In addition, road traffic PM_2.5_ was 35 ± 6 µg/m^3^ on average; a similar value of PM_2.5_ from traffic was reported by Abera (2020) in Adama-Addis Ababa (33 µg/m^3^), a lower value by Belarbi (2020) in Algeria (19.71 µg/m^3^), and a higher value was reported by Ariunsaikhan (2020) in Mongolia (42,871 µg/m^3^) [29,30,31]. Cooking represented 41 ± 1 µg/m^3^, and a similar value of PM_2.5_ from cooking was reported by Vliet al. (2013) in Ghana (46.6 µg/m^3^) [12].

The DRI group displayed the highest daily averaged concentration (excluding periods involving usage of IST and ICS) of 27 µg/m^3^, followed by COOK (24 µg/m^3^), ST (18 µg/m^3^), and OW (12 µg/m^3^). The higher values for DRI and COOK were attributed to high PM_2.5_ concentrations during daytime activities of driving and cooking, respectively. On the other hand, the low daytime PM_2.5_ concentrations in offices and schools resulted in lower daily values for OW and ST, as shown in Table 1. However, when concentrations with IST/ICS at home were involved in the daily average estimation, the daily average concentration increased significantly. The concentration increased from 20 to 54 µg/m^3^ (Table 1), which was twice as large as the WHO guideline. OW and COOK participants showed similar average concentrations of IST/ICS of 244 and 300 µg/m^3^, respectively. In both groups, the participants reported the simultaneous use of IST and ICS in the diaries. Drivers presented lower values, which was attributed to the use of only IST in a microenvironment located near the participants’ rooms. The group of students had the lowest concentration associated with the combustion of ICS only in the house.

**Figure 5:**
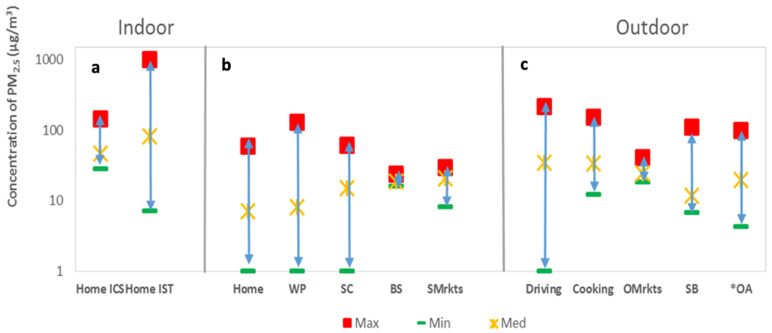
Average maximum (Max), minimum (Min), and median (Med) concentrations for different activities and microenvironments indoors (**a**,**b**) and outdoors (**c**). IST: Insecticide, ICS: Incense, WP: Workplace, SC: School (classes), BS: Beauty salon, Smrkts: Supermarkets, Omrkts: Open markets, SB: School (break), OA: Other activities. * Activities recorded indoors and outdoors.

Figure 5 displays the estimated average maximum, minimum, and median PM_2.5_ exposure concentrations indoors (a,b) and outdoors (c). The PM_2.5_ maximum average concentrations reached very high levels for all indoor and outdoor activities and microenvironments. The highest average maximum and median values were attributed to indoor exposure (IST/ICS) and outdoor exposure (driving and cooking). The highest level of 999 µg/m^3^ was observed indoors during the combustion of IST, which is consistent with results presented by Manigrasso et al. where indoor PM emissions were the highest while combustion sources, such as mosquito coil and incense coil, were present [32]. The second highest was attributed to driving (216 µg/m^3^), followed by cooking at a concentration of 150 µg/m^3^. The use of only ICS at home represented another high-exposure microenvironment, with an average maximum exposure of 145 µg/m^3^. The highest median exposure occurred during special events at 82 and 46 µg/m^3^, respectively, for IST and ICS. Driving and cooking were 34 µg/m^3^. Fifty percent of the exposure concentration was higher than the median values. For most activities and microenvironments, the median concentration exceeded the WHO daily standard of 25 µg/m^3^ [33]. The average minimum concentrations were ≤14 µg/m^3^, depending on the activity and microenvironment.

The indoor PM_2.5_ concentration did not exceed 11 µg/m^3^ without the combustion of the specific products. Therefore, indoor exposure could significantly decrease with a decrease in the use of IST/ICS. The outdoor average concentration was as low as 9 µg/m^3^ in the neighborhood, while the concentration in the yards of houses reached 22 µg/m^3^ (Table S1). For both indoor and outdoor environments, background concentrations were lower than the observed concentrations during anthropogenic activities. This implies that in Bamako, anthropogenic activities are the most likely factor of exposure, rather than outdoor elevated sources.

## 4. Discussion

### 4.1. Identification of Activities Producing Greatest Exposure to PM_2.5_

**Figure 6:**
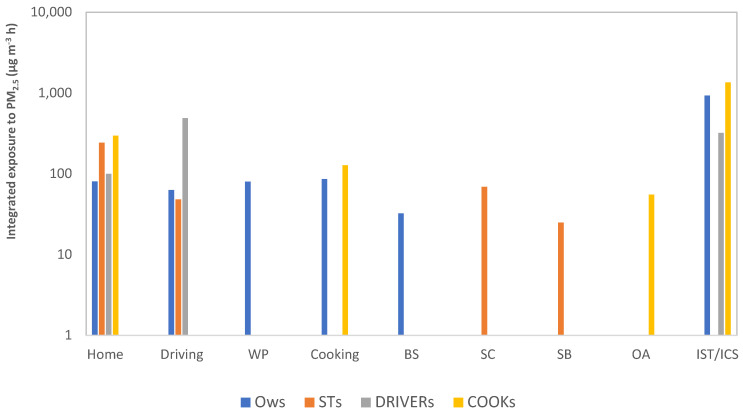
Integrated PM_2.5_ exposure for different activities. IST: Insecticide, ICS: Incense, STs: Students, OWs: Office workers, WP: Workplace, BS: Beauty salon, SC: School (classes), SB: School (break), OA: other activities.

Total integrated exposure, which is the product of concentration and exposure time, was calculated for each group to characterize the exposure during different common daily activities (Figure 6).

The integrated exposure varied in different microenvironments, depending on the activity and its duration. Special events were frequent and represented the highest integrated exposure for OW, COOK, and DRI (927, 1350, and 320 µg m^−3^ h, respectively). The combustion of IST/ICS is a common practice in households in a wide area (Africa, Asia, and South America). Although indoor air pollution has been widely addressed in the literature, the use of IST/ICS has received comparatively little interest.

Highly integrated exposures were also observed during traffic (driving and commuting). The DRI group with a value of 487 µg m^−3^ h presented the highest integrated exposure, followed by OW and ST groups (63 and 48 µg m^−3^ h, respectively).

Cooking represented another activity with high integrated exposure values of 127 and 86 µg m^−3^ h in the COOK and OW groups, respectively. In this study, the COOK group presented a significantly high integrated exposure of 432 µg m^−3^ per day compared to the one presented by Vliet et al. (2013) in rural Ghana of 128.5 µg m^−3^ per day [12]. This marked difference might be attributed to the additional daily activities performed by cooks in the urban city of Bamako, such as commuting, cleaning, and grocery acquisition.

**Figure 7:**
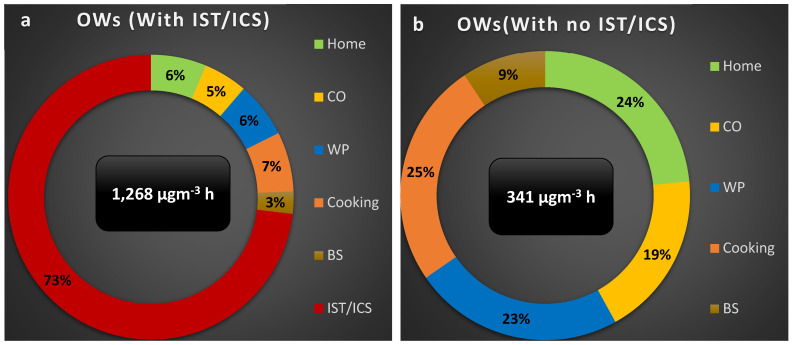
Percentage of integrated exposure for different activities and microenvironments for office workers including (**a**) and excluding (**b**) the combustion of IST/ICS. OW: Office worker IST: Insecticide, ICS: Incense, CO: Commute, WP: Workplace, BS: Beauty salon.

Additionally, the percentages of integrated exposure attributed to different microenvironments were estimated. The value of the integrated exposure for each microenvironment was divided by the sum of the integrated exposure of all the microenvironments in each group. Figure 7 presents the percentages of the integrated exposure for the OW group with and without the use of specific products (a and b, respectively). Percentages for the other groups are given in the Supplementary Materials (Figures S7–S9). For the OW group, the highest percentage of integrated exposure was attributed to the use of IST/ICS (73%, Figure 7a). Without special events, the highest percentage was attributed to cooking (Figure 7b). The data in Figure 7a,b indicate the total integrated exposure decreased by approximately a factor of 4, from 1268 to 341 µg m^−3^ h, without the combustion of IST/ICS. PM emissions from fossil fuel and biofuel combustion are expected to increase significantly in Africa in the near future [34]. An increase of 10 µg/m^3^ of PM_2.5_ is associated with high respiratory disease mortality and morbidity of 4.6% and 4.5%, respectively [5]. Even though houses in Bamako have natural ventilation systems, the windows and doors are usually kept closed while using products, such as IST and ICS.

**Figure 8:**
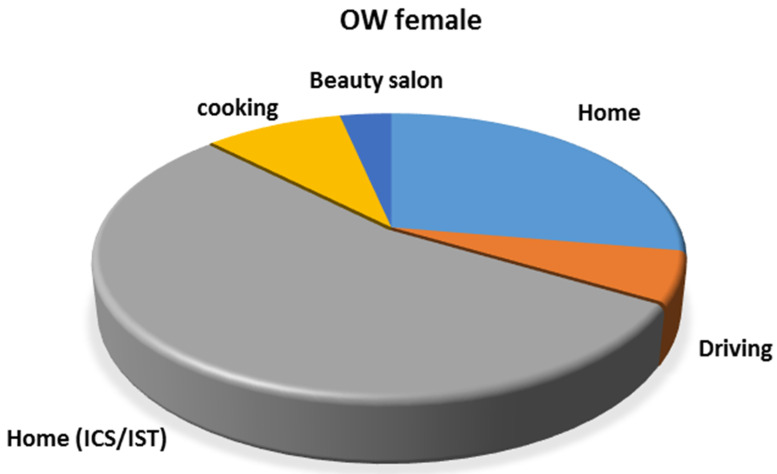
Gender-related integrated exposure to PM_2.5_ adopted from OW5 on 26 and 27 September 2020. OW: Office worker; IST: Insecticide; ICS: Incense.

In Bamako, occupational gender segregation influences the degree of the integrated exposure. All public transportation drivers are men, and cooks are women. According to the WHO, emissions from road traffic have been linked to a wide range of health effects, including effects on the cardiovascular and respiratory systems [35]. Public transportation drivers spend more than 10 h per day working (Figure 4c). Thus, they are subject to chronic exposure. Moreover, domestic biomass usage for cooking is one of the major PM_2.5_ exposure sources in developing countries [36]. Associated with domestic and forest fires, cooking has generated global concern for its effects on human health and the environment [37]. In 2010, the use of solid fuels for household cooking resulted in 370,000 deaths and 9.9 million disability-adjusted life years on a global scale [38]. In Mali, wood and charcoal represent more than 75 percent of the household energy needs [16]. Solid fuel is usually combusted in inefficient cooking stoves, producing a variety of health-damaging particles [38]. The effects of air pollution can be observed during pregnancy [39]. Chronic exposure to PM_2.5_ concentrations exceeding 30 µg/m^3^ has been associated with maternal death [9]. Globally, emissions from households’ solid fuel burning represented the second largest health risk factor in women, and the third in children on a global scale [12,22]. In Bamako, children spend a considerable amount of time with their mothers during the first few years of their lives. Nevertheless, women are the most exposed to PM from cooking, suggesting that children are subject to PM exposure from cooking. In addition, unlike males, female OWs perform other activities, especially during weekends, exposing them to supplementary sources of PM_2.5_. Figure 8 shows the gender-related integrated exposure for a female OW. In addition to working, female office workers often cook and take care of the house; therefore, they can be exposed to supplementary PM_2.5_ sources.

The findings allowed us to identify three main common daily activities exposing the population to high concentrations of PM_2.5_: combustion of specific products (IST and ICS) in households, traffic, and cooking.

### 4.2. Comparison of PM_2.5_ Concentration with WHO Standards and Health Effects

The 24 h average concentrations with and without the use of the specific products were compared to the WHO standards. With no ISC/ICS, DRIs and COOKs presented very close values to the WHO’s guideline for 24 h exposure concentration of 25 µg/m^3^ [33]. However, OWs and STs displayed lower values than the guideline (Table 1). Concentrations were markedly increased in the OW, COOK, and DRI groups with the use of specific products. Concentrations were 2, 1.5, and 3 times higher, respectively, than the recommended daily limit. Diverse health issues from exposure to high PM_2.5_ concentrations have been mentioned in many studies. For example, Li et al. (2013) [40] reported concentrations exceeding 20 μg/m^3^ as the most harmful to human respiratory health. In the present study, all four groups exceeded the yearly limits of 10 µg/m^3^ with no use of IST/ICS. After considering the use of specific products, the ST, DRI, OW, and COOK groups presented values that were 2, 4, 5, and 7.5 times higher, respectively, than the yearly recommended limit of exposure to PM_2.5_. Burning one mosquito coil can release the same amount of PM_2.5_ as burning 75–137 cigarettes [41]. The combustion of IST has been associated with toxicological effects, morphological alterations, and enzyme perturbations [41]. Asthma, lifetime eczema, wheezing, and rhinitis have been associated with the combustion of IST. ICS burning has been found to be associated with wheezing and rhinitis [42]. Furthermore, from previous studies, the combustion of IST/ICS was found to liberate compounds that are carcinogenic to humans [32].

In Bamako, the incidence of acute respiratory disease increased from 282,000 cases in 2001 to more than 1,500,000 in 2016 [18]. An annual concentration of 10 µg/m^3^ is recommended as the long-term exposure limit. However, a recent study pointed out the effects of long exposure to PM_2.5_ concentrations below the recommended limits [43]. Disease from air pollution results in a decrease in life expectancy, similar to those of other high-priority risk factors and diseases [44].

## 5. Conclusions

Relationships between personal exposure to PM_2.5_ and daily activities were investigated in the city of Bamako using palm-sized optical PM_2.5_ sensors. Sampling was performed indoors and outdoors to determine the exposure level for different activities and microenvironments. The participants were divided into four groups according to their main occupation. Three daily activities that highly expose the population to PM_2.5_ have been identified as the most exposed inhabitants.

The study revealed that Bamako’s inhabitants are highly exposed indoors while combusting IST and ICS, where concentrations reached 999 µg/m^3^ on average in a 10 min period. Likewise, participants were least exposed indoors when IST/ICS were not used. Traffic and cooking were identified as high-exposure activities. The concentrations fluctuated among the groups according to their main occupation. Public transportation drivers and cooks were most exposed to PM_2.5_ during their daily activities. Comparisons of our results with the WHO standards indicated that all participants exceeded the yearly exposure limits. Office workers and students presented values within the daily exposure limit without the use of specific products. On the other hand, the group of cooks and drivers exceeded this limit. Although the concentration in students was within the daily limit, the young age makes them more susceptible to air pollution.

Observing the populations actions and avoiding activities that increase pollution could help reduce pollution levels, and consequently, reduce exposure levels. An increase in the level of PM_2.5_ leads to negative effects on health, especially for sensitive people [45]. According to Manigrasso et al. (2017), the impact of particulate matter is higher for sensitive people compared to healthy individuals [46]. Indoor air quality is highly critical for health, since people typically spend most of their time indoors. The use of specific products indoors greatly increased the concentration of PM_2.5_. Therefore, reducing the use of these products could reduce the exposure at specific locations and reduce the risk level of individual total inhaled doses [47]. Moreover, Vliet et al. (2013) suggested that a change in individual behavior could help reduce exposure to PM_2.5_ [12]. Communities, once aware of the potential health effects from their daily indoor actions, can manage their own exposure by avoiding or reducing activities that emit pollution.

This is the first study assessing personal exposure to PM in the city of Bamako. The results provide valuable information about the level of exposure. The results indicated that the main exposure sources were related to lifestyle, IST/ICS use, driving, and cooking. The exposure levels were acute and, the emission sources were strongly related to daily anthropogenic activities rather than natural emissions. This indicates the advantage of personal exposure monitoring.

This study could help increase populations’ awareness and should be useful for decision-makers. Consequently, it could help achieve SDG goals, especially regarding health and the environment. For instance, reducing personal exposure to PM_2.5_ through individual daily actions could reduce health impacts and thus contribute to the SDG3 on health and wellbeing. Furthermore, improving the quality of fuel used by strengthening laws on the fuel quality, using catalytic converters to the vehicle exhausts, reinforcing laws on the annual vehicles’ technical inspections, promoting teleworking, and the shift in working hours for different institutions and companies could significantly reduce road traffic-induced pollution. Indeed, teleworking can moderate traffic congestion, thus improving air quality and facilitating urban planning and development [48]. Gradually switching from the use of charcoal and wood to the use of cleaner energies, such as solar energy and biogas, could alleviate the exposure to PM_2.5_ from biofuel combustion, as well as pollution from waste incineration. In particular, countries like Mali have high solar potential energy and face waste management issues. These alternatives are economically challenging for developing countries and require time, as proper planning and funds are needed. Therefore, promoting the use of improved cook-stoves with higher combustion efficiency could be practical as a transitional state. Adopting such strategies could help mitigate urban air pollution in Bamako and help achieve SDG7 on affordable and clean energy and SDG13 on climate change at different scales. There is a strong connection between global and local environmental concerns. Although many urban issues are confined within the local scale, many others have regional, or even global consequences. Reducing local emissions has positive repercussions on the mitigation response to regional and global environmental issues, as emissions typically originate from local sources [49].

Our findings demonstrate the need to design appropriate control strategies and continuous monitoring of PM_2.5_, to reduce emissions and protect public health. More work is needed to obtain information on the chemical composition of PM_2.5_, and hence, more information on emission sources and specific health effects in this region.

## Figures and Tables

**Table 1:** Summary of PM_2.5_ concentrations (µg/m^3^) during the participants’ daily activities. Numbers in parentheses show exposure time (hours).

	OW	ST	DRI	COOK	Average
**Home**	9	14	12	18	13 ± 4
**Driving/commute**	33 (1.9)	30 (1.6)	42 (11.6)		35 ± 6
**Workplace**	14 (5.7)				
**Cooking**	43 (2)			41 (3.1)	42 ± 1
**Beauty salon**	19 (1.7)				
**School (classes)**		16 (4.3)			
**School (Break)**		31 (0.8)			
**Home (IST/ICS)**	244 (3.8)	30 (2.6) *	78(4.1)	300 (4.5)	207 ± 115 **
**Daily average**	12	18	27	24	20 ± 7
**Daily average (IST/ICS)**	49	20 *	38	76	54 ± 20 **

* STs were exposed only to ICS and not to IST. ** ST was excluded.

## Data Availability

Data sharing is not applicable to this article.

## Supplementary Materials

The following supporting information can be downloaded at: www.mdpi.com/article/10.3390/ijerph19010611/s1, Figure S1: (a) Daily mass concentration variation of PM_2.5_ for the Yamashina versus P-sensor. (b) Correlation plots of the P-sensor versus GRIMM OPC; daily average concentration; Table S1: Average concentration for different daily indoor and outdoor activities; Figure S2: Yoshida south campus information; Table S2: Personal information collection sheet for participants; Figure S3: Insecticide and incense; Figure S4: Traffic situation in Bamako city; Figure S5: Cooking facilities in Bamako; Figure S6: Image of school situation; Table S3: Summary of PM_2.5_ concentrations (µg/m^3^) during daily activities of office workers; Table S4: Summary of PM_2.5_ concentrations (µg/m^3^) during daily activities of students; Table S5: Summary of PM_2.5_ concentrations (µg/m^3^) during daily activities of cooks; Table S6: Summary of PM_2.5_ concentrations (µg/m^3^) during daily activities of drivers; Figure S7: Percentage of integrated exposure for different activities and microenvironments for students, Including (a) and excluding (b) the combustion of IST/ICS; Figure S8: Percentage of integrated exposure for different activities and microenvironments for drivers, including (a) and excluding (b) the combustion of IST/ICS; Figure S9: Percentage of integrated exposure for different activities and microenvironments for cooks including (a) and excluding (b) the combustion of IST/ICS.
