# Supplementary material for: Personal Exposure to Fine Particles (PM2.5) in Northwest Africa: Case of the Urban City of Bamako in Mali
Source: Int J Environ Res Public Health. 2022 Jan 5;19(1):611. doi: 10.3390/ijerph19010611 (PMC8744751; doi:10.3390/ijerph19010611)
Supplement: Supplementary file 1 [file ijerph-19-00611-s001.zip › ijerph-1446385-supplementary.pdf]

Personal Exposure to Fine Particles ($PM_{2.5}$) in Northwest Africa: Case of the Urban City of Bamako in Mali

[1] Graduate School of Global Environmental Studies, Kyoto University, Yoshida-Honmachi, Sakyo-ku,
Kyoto 606-8501, Japan; sakamoto.yosuke.7a@kyoto-u.ac.jp (Y.S.); murano@hosei.ac.jp (K.M.); kajii.yoshizumi.7e@kyoto-u.ac.jp (Y.K.)
[2] Graduate School of Human and Environmental Studies, Yoshida-Nihonmatsu-Cho, Sakyo-ku,
Kyoto 606-8501, Japan
[3] Regional Environment Conservation Division, National Institute for Environmental Studies, Tsukuba 305-8506, Japan
[4] Laboratoire de Biologie Moléculaire Appliquée, Faculté des Sciences et Techniques (FAST), University of Sciences, Techniques and Technologies of Bamako (USTTB), Bamako E 3206, Mali;
okoita@icermali.org (O.A.K.); ibrahim.traore@lbma.edu.ml (I.T.); yacoubadansoko@yahoo.fr (Y.D.)
* Correspondence: alimatasidibe93@gmail.com or sidibe.alimata.32x@st.kyoto-u.ac.jp

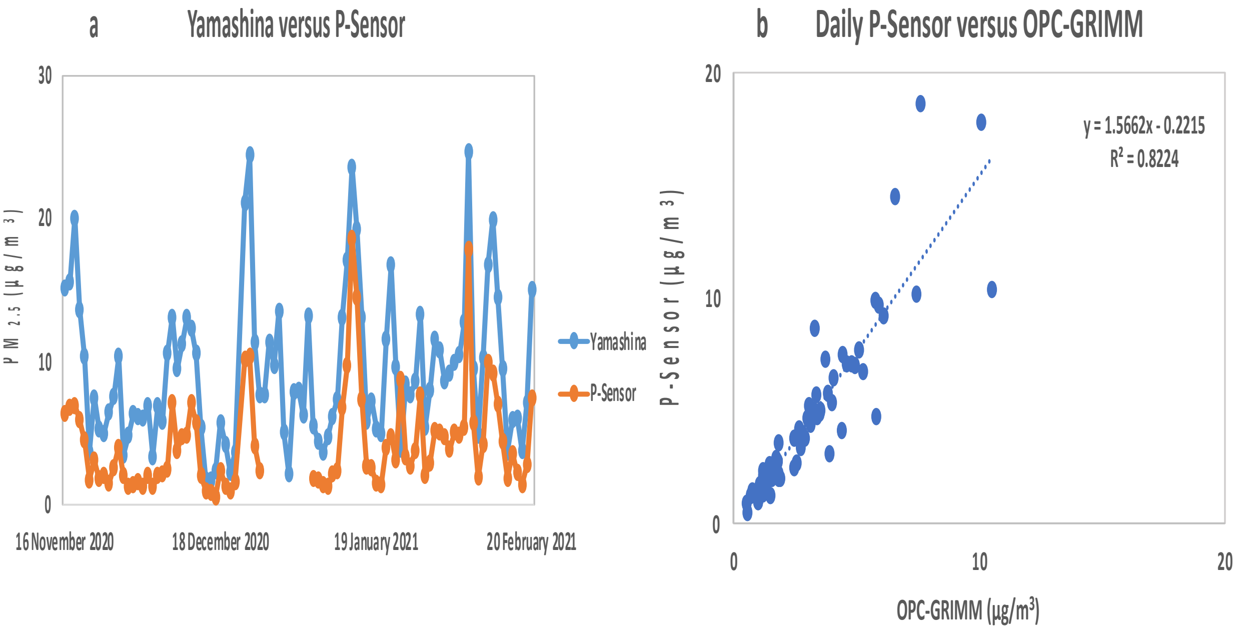

Figure S1. (**a**) Daily mass concentration variation of $PM_{2.5}$ for the Yamashina versus P-sensor. (**b**) Correlation plots of the P-sensor versus Grimm OPC from 16 November to 22 December 2020, and from 7 January to 20 February 2021; daily average concentration.

Figure S1 shows a comparison between the $PM_{2.5}$ sensor (P-sensor) and data from a local monitoring station, the Yamashina station of the Japanese Ministry of Environment (AEROS), which is located at 5.8 km southeast away from the Yoshida campus of Kyoto university.

Table S1. Average concentration for different daily indoor and outdoor activities.

Activities/$PM_{2.5}$ Concentration, μg/m³					
Indoor	Bedtime (insecticide)	267	Outdoor	Cooking	41
	Bedtime (no insecticide)	7		2 h after cooking	30
	Home (incense)	75		Market	25
	Home (no incense)	11		Yard	22
	School	18		Driving	39
	office	19		Walking neighborhood	9
Others					24

This table shows the average concentration of $PM_{2.5}$ (μg/m³) in different microenvironments during different daily activities for the overall samples. The indoor $PM_{2.5}$ concentration did not exceed 11 μg/m³ without the combustion of the special products. The outdoor average concentration was as low as 9 μg/m³ in the neighborhood, while the concentration in the yards of houses reached 22 μg/m³.

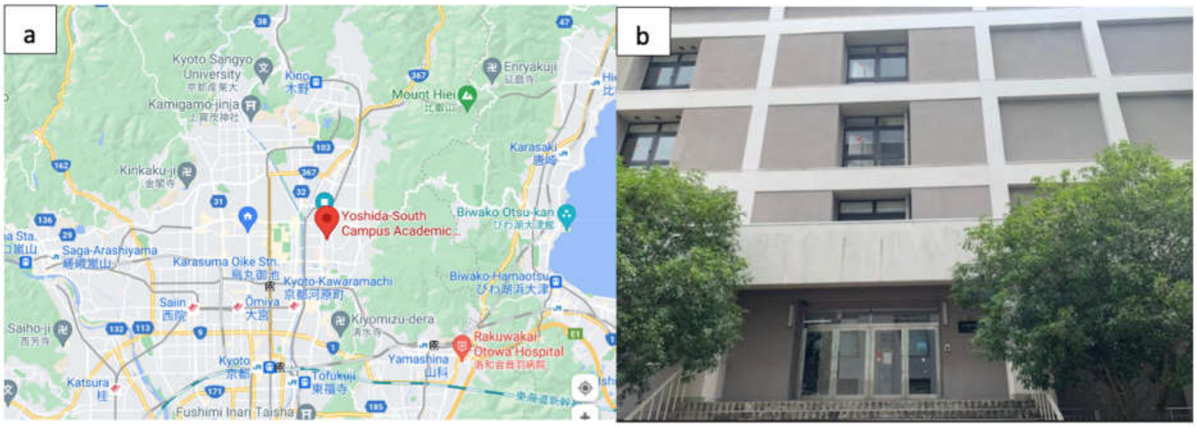

Figure S2. Yoshida south campus information. (**a**) Location of the sampling site. (**b**) Image of the building.

Table S2. Personal information collection sheet for participants.

**Groupe**							**Date**				
**Numéro de capteur**	**Nom**	**Prénom**	**Age**	**Sex**	**Occupation**	**Quartier de résidence**	**Lieu de travail/ d'étude**	**Moyen (s) de transport**	**Fumeur/non-fumeur**	**Activité(s) sportive (s)**	**Numéro de téléphone et/ou mail**	Signature

This table was used to collect personal information about the volunteers participating in the personal exposure assessment sampling.

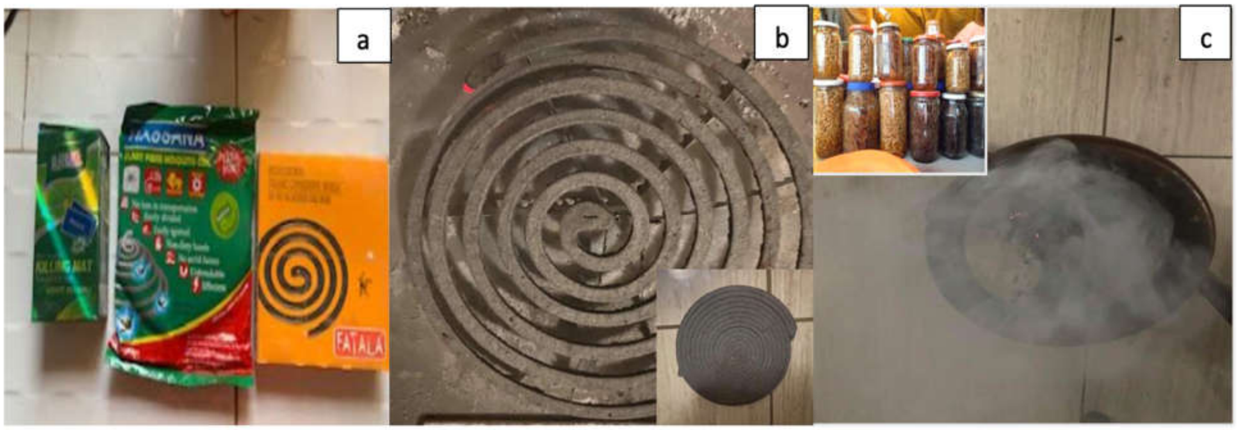

Figure S3. Insecticide and incense. (**a**) The three most popular insecticides used in Bamako. (**b**) Combustion of insecticide-mosquito coils. (**c**) Combustion of incense.

The pictures above show the special products used in households in Bamako. (a) The three types of mosquito coils that are widely used in Bamako. They are usually burned slowly (b) or using traditional burners (c). Panel (c) shows the traditional incense in glace jars and the burning condition using a traditional burner (woussoulan bele).

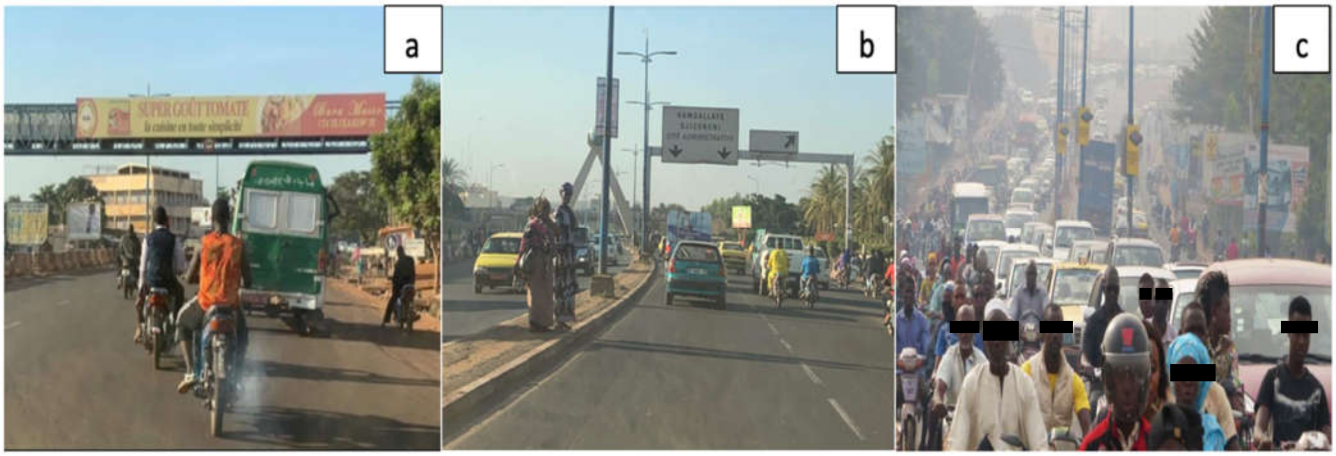

Figure S4. Traffic situation in Bamako city. (**a**,**b**) Photo credit for Dienta Alimata. (**c**) Journal scientific et technique du Mali, 2018.

Figure S4 shows pictures of the real-life traffic situation in Bamako. Panel (c) shows periods of road congestion times. This generally occurs during rush hours when workers and students commute from home to work or school and during their lunch break.

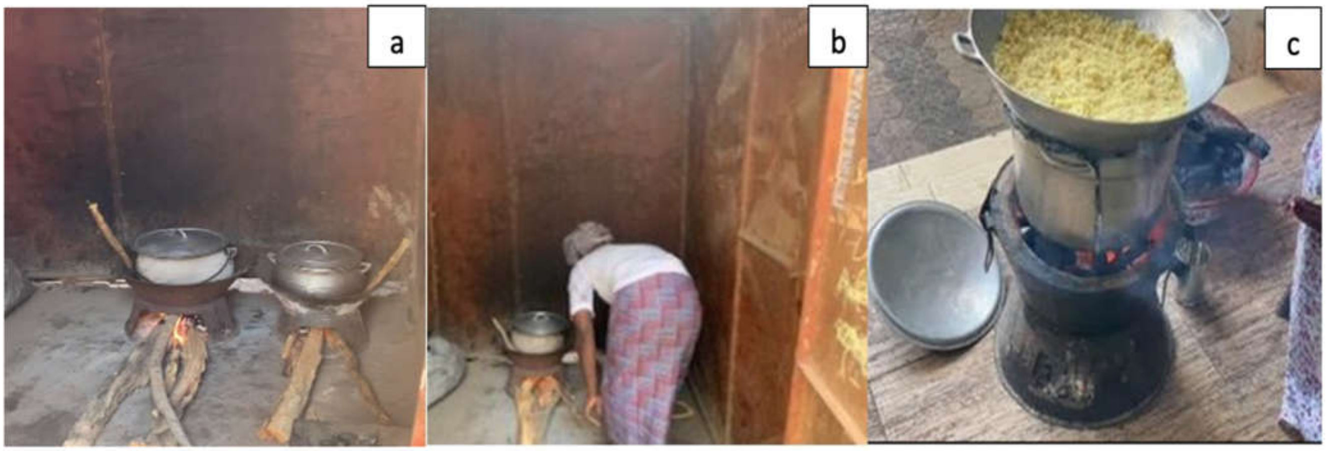

Figure S5. Cooking facilities in Bamako: (**a**,**b**) wood and (**c**) charcoal.

Figure S5 gives an idea of the cooking conditions in Bamako. Wood and charcoal are the two main energies used for cooking, and they are combusted in traditional furnaces. Figure S5b shows a cook rearranging the wood for cooking.

Students from the school Ke Moussa Dienta in Bamako-Mali participated in this study. Figure S6 shows images of the school's situation and students taking classes.

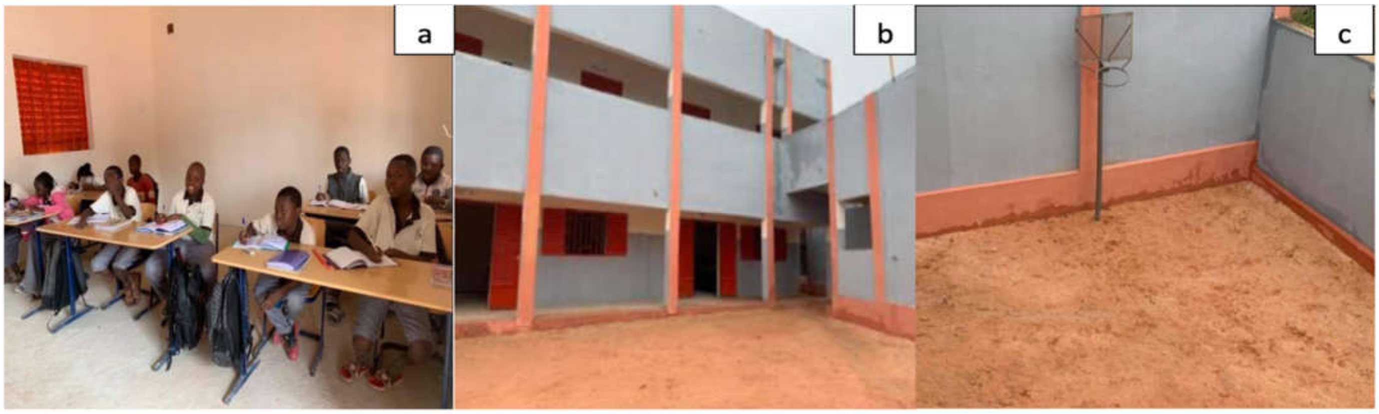

Figure S6. Image of school situation: (**a**) students in class group 2, (**b**,**c**) schoolyard.

Table S3. Summary of $PM_{2.5}$ concentrations (μg/m³) during daily activities of office workers. Numbers in parentheses show the exposure time (hours). OW: Office worker; ISC: Incense; IST: insecticide.

		Home	Driving	Work Place	Home (IST/ICS)	Cooking	Beauty Salon	Daily Average
OW1	3 September	10	28 (1.0)	5 (6.0)				10
	4 September	10	28 (1.3)	5 (4.8)				10
	5 September	10	28 (0.8)	4 (5.2)				9
OW2	8 October	5	30 (0.3)	33 (10.3)				17
	9 October	11	66 (1.0)	48 (4.7)				20
	10 October *	9	16 (4.3)					
OW3	14 September	5	44 (2.7)	4 (3.5)				9
	16 September	6	23 (1.0)	7 (9.5)				7
	17 September	7	26 (3.0)	7 (5.7)				9
OW4	24 September	7	48 (3.3)	5 (7.0)	305 (4.3)			66
OW5	26 September *	18			160 (3.2)	43 (2.0)		
	27 September *	9	32 (1.7)				19 (1.7)	
Activity average		9	33 (1.9)	14 (5.7)	244 (3.8)			

* These days involve data for a part of the day.

All participants had similar daily patterns involving approximately 2 h of driving and approximately 6 h of office work. With only a few exceptions, participants had similar exposures during the same activity. OWs were exposed to approximately 10 μg m^{-3} on a daily average, as seen for OW1, OW2, and OW3. However, their average exposure increased to 66 μg m^{-3} with the use of IST/ICS (OW4). The air condition in the workplace also affected daily exposure. OW2 had a worse condition in the workplace with over 30 μg/m³, which increased the daily exposure of OW2 to twice that of OW1 and OW3.

Table S4. Summary of $PM_{2.5}$ concentrations (μg/m³) during daily activities of students. Numbers in parentheses show exposure time (hours). ST: Student, ICS: Incense, IST: Insecticide.

		Home	Commuting	School	School (Break)	Home ** (ICS/IST)	Daily Average
ST1	16 November	11	32 (0.3)	19 (0.3)			14
	17 November	12	31 (0.5)	17 (5.9)	64 (0.8)	35 (1.25)	16
	18 November	7	9 (0.2)	15 (2.3)			10
ST2	10 November	24	42 (1.9)	20 (6.2)	26 (0.5)		24
	11 November	32	29 (0.8)	6 (3.8)	8 (1.5)		25
	12 November	15	27 (0.2)	21 (5)	52 (0.6)		17
ST3	30 January*	9	28 (7.3)			22 (2)	
	31 January*	13				35 (5.2)	
	1 February	14		12 (6.3)		20 (2)	14
Time average		14	30 (1.6)	16 (4.3)	31 (0.8)	29 (2.6)	

* These days involve data for a part of the day. ** STs were exposed only to ICS but not to IST.

In the ST group, daily concentration was affected by the level of $PM_{2.5}$, during the commute and break time. For ST1, higher concentrations were observed on November 16 and 17, with average concentrations of 32 and 31

μg/m³, respectively, compared to November 18, with an average concentration of 9 μg/m³. Furthermore, on 17 November, the daily average concentration was the highest due to the increase in concentration during the break time. ST2 had a higher concentration at home, resulting in an increase in the daily concentration compared to ST1 and ST3.

Table S5. Summary of $PM_{2.5}$ concentrations (μg/m³) during daily activities of cooks. Numbers in parentheses show exposure time (hours). ICS: Incense, IST: Insecticide.

		Home	Cooking	Home (ICS/IST)	Daily Average
COOK1	25 October	29	47 (5.3)	217 (3)	56
	26 October	26	43 (3.7)	315 (10)	147
	27 October		43 (3.5)	409 (6.8)	140
COOK2	24 October*				
	25 October *	21		880 (2.2)	
	26 October	21	43 (2.8)	153 (5)	48
COOK3	14 January		21 (0.8)		16
	15 January	14	35 (3.7)	37 (0.7)	20
	16 January	8	28 (1.8)	38 (3.8)	23
Time average		18	41 (3.1)	300 (4.5)	

* These days involve data for a part of the day.

As shown in Table S5, all participants showed similar average concentrations recorded at home or during cooking. The concentration of $PM_{2.5}$, during the use of insecticide, considerably increased the daily average concentration. For COOK1, the daily average concentration increased more than two times from October 25, with a concentration at home (ICS/IST) of 217 to 26 and 27 October, with 315 and 409 μg/m³, respectively. In addition, COOK3 showed a lower daily average concentration than COOK1 and COOK2, with a higher average daily concentration at home. The higher the insecticide concentration, the higher was the daily average exposure.

Table S6. Summary of $PM_{2.5}$ concentrations (μg/m³) during daily activities of drivers. Numbers in parentheses show exposure time (hours). DRI: Driver, ICS: Incense, IST: Insecticide.

		Home	Driving	Home (IST/ICS)	Daily Average
DRI 1	11 October	9 (2.7)	36 (12)		16
	12 October	17 (1.5)	43 (12.5)		22
	13 October	4 (4)	41 (12.2)		17
DRI 2	15 November	18 (2.3)	37 (10.8)		18
	16 November	27 (5.7)	55 (11.8)		32
	17 November	20 (2.5)	37 (12)	94 (9.5)	58
DRI 3	30 January	12 (4.5)	40 (12.5)	25 (3)	26
	31 January	12 (1.7)	47 (10.8)	139 (1.7)	47
	1 February	12 (2.2)	45 (10.2)	35 (2.2)	27
Time average		12 (4.1)	42 (11.6)	78 (4.1)	

All participants showed similar daily activity trends with more than 10 h of driving per day and 42 μg/m³ as a daily average (Table S6). Furthermore,

the IST increases the daily average concentration. DRI1 showed a lower daily average compared to DRI2 (November 16 and 17) and DRI3 with IST/ICS recorded.

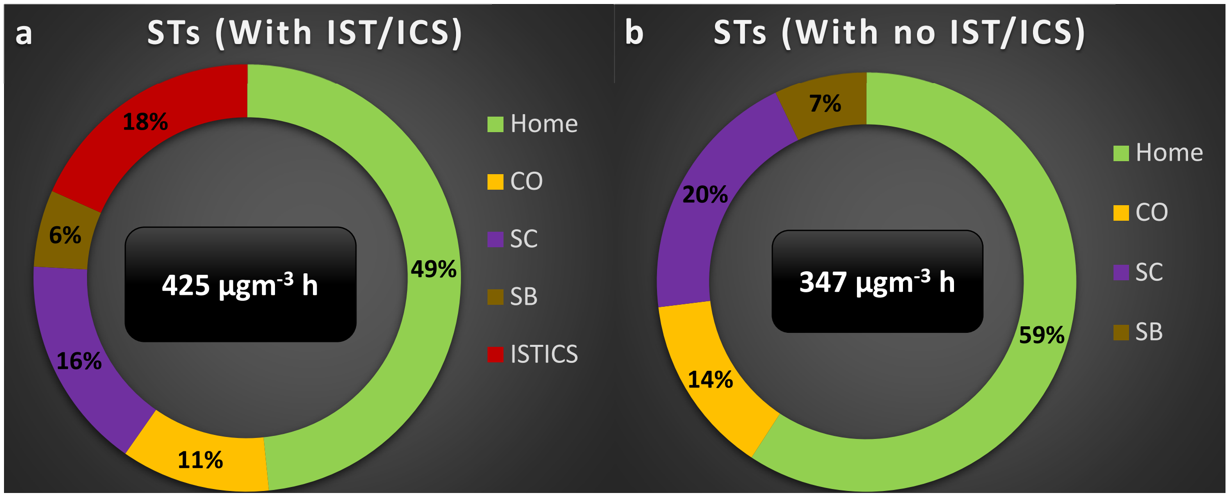

Figure S7. Percentage of integrated exposure for different activities and microenvironments for students, including (**a**) and excluding (**b**) the combustion of IST/ICS. ST: student, CO: Commute, IST: Insecticide, ICS: Incense, SC: School (classes), SB: School (break time).

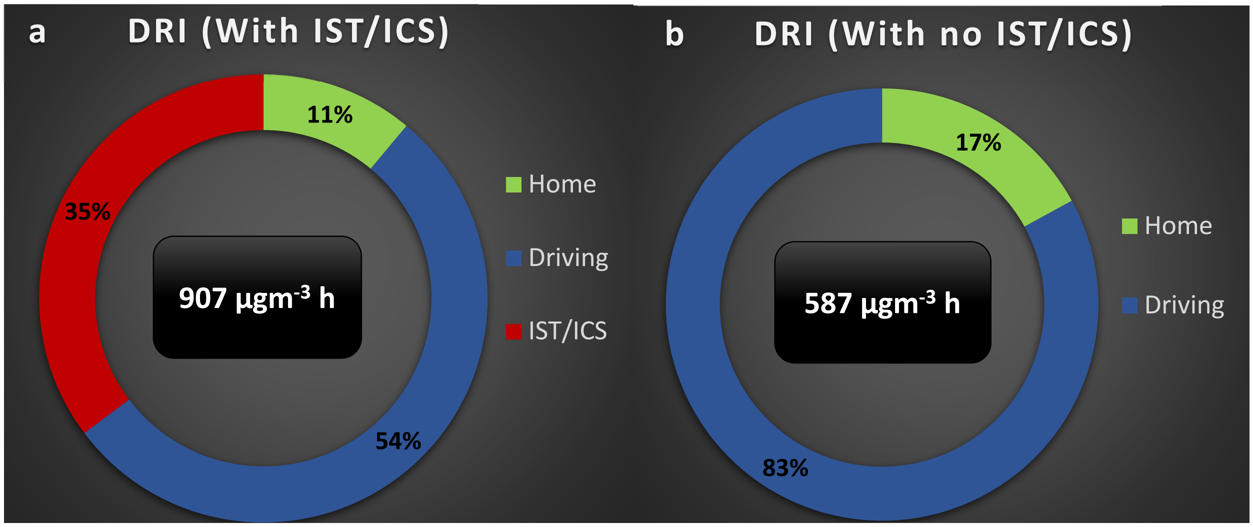

Figure S8. Percentage of integrated exposure for different activities and microenvironments for drivers, including (**a**) and excluding (**b**) the combustion of IST/ICS. DRI: Driver, IST: Insecticide, ICS: Incense.

For students, there was no significant change in the percentages from the use to the non-use of special products. For drivers, the highest percentage was observed during working hours (driving). IST/ISC showed a lower percentage due to the combustion in another microenvironment than the one the participants stayed in. The total exposure decreased by a factor of approximately 2 and 1 for the DRI and ST groups, respectively, because of the use of only IST in a closed microenvironment for the group of drivers,

and only ICS for students. For the same reason, in these groups, the total exposure decreased by a factor of (approximately) 2 and 1 instead of 4 in the other groups.

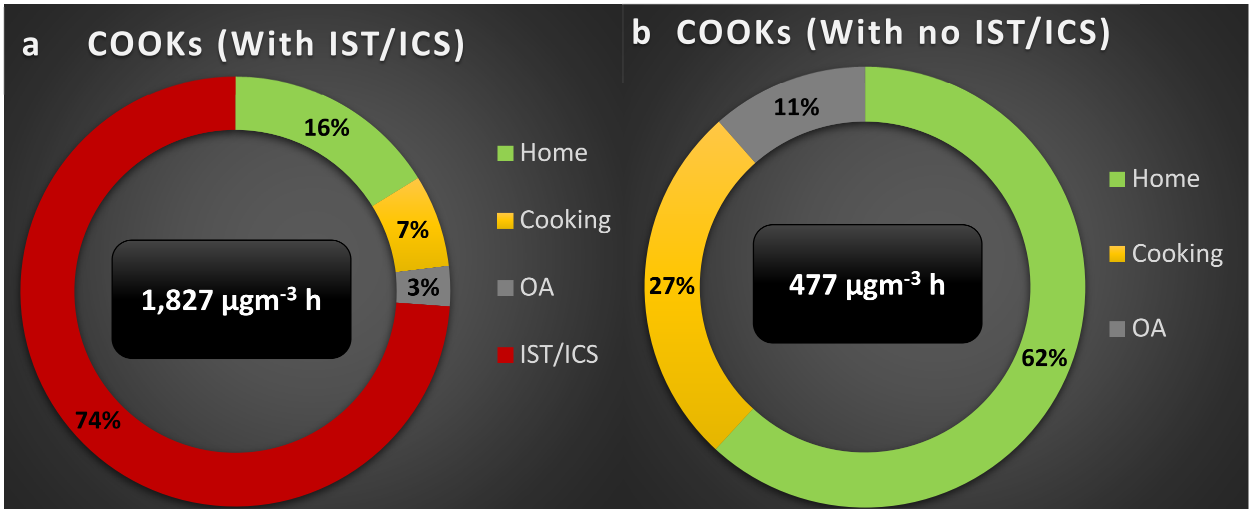

Figure S9. Percentage of integrated exposure for different activities and microenvironments for cooks including (a) and excluding (b) the combustion of IST/ICS. IST: Insecticide, ICS: Incense, OA: Other activities.
